# Associations Between QuantiFERON-TB Gold Plus IFNγ Concentrations and Progression to Symptomatic Tuberculosis in Global High-Burden TB Settings

**DOI:** 10.1093/ofid/ofag437

**Published:** 2026-07-24

**Authors:** Justine Sunshine, Michael Shaffer, Linda L Han, Deepali Gaikwad, Amelia A Houana, Maria Tarcela Gler, Sri Rezeki Hadinegoro, Willem A Hanekom, Javier R Lama, Monde Muyoyeta, Sissy Musala, Videlis Nduba, Valeria C Rolla, Tapash Roy, Jayne S Sutherland, Celso Khosa, Anne Wajja, Timothy M Walker, Amy Cinar, Alexander C Schmidt, Alemnew F Dagnew, Nicole Frahm, for TBV02-E01 Study Group, Q Bhorat, M Loveday, C Duran Palma, J Lombaard, S Cossie, J G Geldenhuys, K Ahmed, E Spooner, L Tina, B Ogutu, R McClelland, W Kilembe, S Kwame, M Inambao, E Sprinz, E Cadena, J R Gonong, E J Berame, M G D Isidro, G Zabat, R G M Veto, Javier R Lama, E Sanchez, V Nankabirwa, H L Nguyen, V N Nguyen, G Marks, B Alisjahbana, E Burhan, N Kaswandani, G Luyeye Matondo Mandiangu, H Z Swah Koko, E Mwamba Kabunda, S Viegas, A L Garcia-Basteiro

**Affiliations:** Clinical Development, Gates Medical Research Institute, Cambridge, Massachusetts, USA; Clinical Development, Gates Medical Research Institute, Cambridge, Massachusetts, USA; Clinical Development, Gates Medical Research Institute, Cambridge, Massachusetts, USA; Department of Pulmonary Medicine, PCMC'S PGI YCM Hospital, Pune, Maharashtra, India; Centro de Investigação em Saúde de Manhiça (CISM), Maputo, Mozambique; TB HIV Innovations and Clinical Research Foundation, Cavite, Philippines; Department of Child Health Faculty of Medicine, University of Indonesia, Jakarta, Indonesia; Africa Health Research Institute, KwaZulu-Natal, South Africa; Asociación Civil Impacta Salud y Educación, Lima, Peru; Centre for Infectious Disease Research in Zambia (CIDRZ), Lusaka, Zambia; National Tuberculosis Program/National TB Reference Laboratory’, Kinshasa City, DRC; Kenya Medical Research Institute, Centre for Respiratory Diseases Research (CRDR), Nairobi, Kenya; Clinical Research Laboratory on Mycobacteria, National Institute of Infectious Diseases Evandro Chagas—Fiocruz, Rio de Janeiro, Brazil; IRD Global, Bangladesh Country Office, Dhaka, Bangladesh; Vaccines and Immunity Theme, Medical Research Council Unit The Gambia at the London School of Hygiene and Tropical Medicine, Fajara, The Gambia; Instituto Nacional de Saúde (INS), Marracuene, Mozambique; Departments of International Public Health and Clinical Sciences, Liverpool School of Tropical Medicine, Liverpool, UK; Department of Physiological Science, Clinical Pharmacology, Faculty of Medicine, Eduardo Mondlane University, Maputo, Mozambique; Medical Research Council, Uganda Virus Research Institute and London School of Hygiene and Tropical Medicine Uganda Research Unit, Entebbe, Uganda; Department of Clinical Research, London School of Hygiene and Tropical Medicine, London, UK; Oxford University Clinical Research Unit, Ho Chi Minh City, Vietnam; Centre for Tropical Medicine and Global Health, Nuffield Department of Medicine, University of Oxford, Oxford, UK; Clinical Development, Gates Medical Research Institute, Cambridge, Massachusetts, USA; Clinical Development, Gates Medical Research Institute, Cambridge, Massachusetts, USA; Clinical Development, Gates Medical Research Institute, Cambridge, Massachusetts, USA; Clinical Development, Gates Medical Research Institute, Cambridge, Massachusetts, USA

**Keywords:** biomarker, IFNγ, IGRA, QFT-plus, tuberculosis

## Abstract

**Background:**

Predictive biomarkers for symptomatic tuberculosis (TB) progression would transform targeted prevention efforts. Although interferon-gamma release assays (IGRAs), including QuantiFERON® TB-Gold Plus (QFT-Plus), have been studied for this purpose, systematic evaluation of the QFT-Plus TB1 and TB2 Interferon-Gamma (IFNγ) concentrations remains limited, particularly in high-burden TB settings.

**Methods:**

Baseline TB1 and TB2 IFNγ concentrations from 5246 participants (ages 15–34 years) in TB-endemic regions were analyzed in relation to subsequent TB outcomes over a median of 525 days follow-up (NCT05190146). Participants were categorized as controls (no TB), suspected TB (no microbiological confirmation), or laboratory-confirmed TB, including a subset meeting a stringent case definition (≥2 positive microbiologic tests). Associations between baseline IFNγ concentrations and progression to symptomatic TB were assessed.

**Results:**

In the full cohort (IGRA+/− participants), baseline TB2 IFNγ concentrations were significantly higher compared with controls among participants who developed suspected TB (*P* = .01), laboratory-confirmed TB (*P* = .01), or met the stringent case definition (*P* < .0001). In IGRA+ participants, baseline TB2 concentrations were significantly higher than controls in suspected (*P* = .01) and laboratory-confirmed (*P* = .02) groups. Associations with baseline TB1 IFNγ concentrations and TB progression were observed for participants meeting the stringent case definition within the full cohort (*P* = .001). Among stringent definition cases, TB2 concentrations achieved an area under the receiver operating characteristic curve of 0.84, with sensitivity of 80% and specificity of 78%.

**Conclusions:**

Quantitative IFNγ concentrations from QFT-Plus, particularly TB2, were associated with progression to symptomatic TB, met or exceeded WHO-recommended sensitivity and specificity thresholds for predictive biomarkers, and may support biomarker-based stratification in TB clinical research.

## Teaser

In an exploratory analysis from a global tuberculosis (TB) epidemiology study (NCT05190146), higher QuantiFERON-TB Gold Plus IFN? concentrations were associated with progression to symptomatic TB and met WHO predictive biomarker thresholds, supporting their potential for risk stratification in TB research.

Tuberculosis (TB) remains one of the most significant global health challenges with more than 10 million cases and over 1 million deaths reported in 2023 [1]. It is estimated that one fourth of the world's population is or has been infected with *Mycobacterium tuberculosis* (*Mtb*). While many infections are believed to be contained or eliminated, ∼5%–10% of individuals infected with *Mtb* will progress to TB disease, with the highest risk of progression within the first 2 years following infection [2]. Targeting individuals at the highest risk of developing active TB with preventive treatment is central to the WHO End TB Strategy [3]. A predictive biomarker for progression to active TB would enable more efficient use of resources by identifying individuals most likely to develop disease, supporting both local care and global elimination efforts. Furthermore, as most current TB vaccine and prevention trials use symptomatic, microbiologically confirmed TB as the primary endpoint, there is also interest in identifying biomarkers that predict progression to symptomatic TB disease in a manner that aligns with a trial's endpoint definition. Such endpoint-matched biomarkers would be valuable in designing efficient, well-powered trials targeting those at highest risk.

The WHO specifies minimum sensitivity and specificity of >75% for tests predicting progression within 2 years, providing an important benchmark for evaluating biomarkers of symptomatic TB progression [4]. Current diagnostic tests, including the commonly used Interferon-Gamma Release Assay (IGRA), do not currently meet this minimum performance criterion [5, 6]. IGRA tests measure immunoreactivity to TB antigens through detection of released interferon-gamma (IFNγ), generally via an Enzyme-Linked Immunosorbent Assay (ELISA). As such, the IGRA test does not measure *Mtb* burden but rather a TB-specific effector or memory immunological response, which indirectly measures prior or ongoing *Mtb* exposure. A frequently used IGRA test is the QuantiFERON® TB-Gold Plus (QFT-Plus), which contains 2 TB antigen stimulation tubes: TB1 and TB2 [7]. The TB1 tube contains peptides derived from TB antigens ESAT-6 (early secreted antigen target 6 kDa) and CFP-10 (culture filtrate protein 10) and is designed to detect TB-specific CD4^+^ T-cell responses [7]. The TB2 tube contains the same peptides as TB1 but with additional shorter peptides designed to detect TB-specific CD8^+^ T-cell responses [7]. Whereas previous iterations of the QFT test (QFT Gold-in-Tube, GIT) only contained the TB1 tube (with additional TB7.7 peptides), the TB2 tube was included in the QFT-Plus test due to emerging literature that showed TB-specific CD8^+^ T-cell responses were associated with increased *Mtb* bacterial load, recent exposure to *Mtb*, and active TB disease [8–10].

While the QFT test qualitative readout (positive/negative/indeterminate) has been shown to be a poor predictive biomarker [11], there has been considerable interest in the evaluation of the quantitative IFNγ readout in predicting progression to TB disease. Most studies of quantitative IFNγ responses used QFT-GIT data and therefore focused on the single antigen tube. A meta-analysis of 37 studies found a concentration-dependent relationship between IFNγ levels and risk of progression to TB disease [12], a finding supported by additional studies in high and low-endemic settings [13, 14]. Notably, across all studies, there was considerable variation in the cohorts as well as substantial overlap in TB-specific IFNγ responses between individuals who do and do not progress to symptomatic TB disease, thus limiting its discriminatory power [12]. In addition, both the QFT-GIT and QFT-Plus tests are prone to considerable analytical variability [15] and thus it is difficult to directly compare results across studies conducted in different laboratories, as changes to sample handling and processing can impact quantitative measurements [16–18]. For the QFT-Plus assay, several studies have affirmed that TB2-specific IFNγ values are higher in individuals with active TB disease and recent TB infection [19–22], but studies have been very limited in evaluating TB1 versus TB2-specific responses in predicting progression to TB disease [13], especially in high-burden countries.

We recently completed a global observational epidemiologic study in 45 sites across 14 countries to evaluate IGRA status among people living in settings of high TB risk [23]. This study was designed to describe both the proportion of IGRA positivity by site and the incidence of suspected and laboratory-confirmed (lab-confirmed) pulmonary TB to inform and build capacity for phase 3 TB vaccine efficacy trials. IGRA status was assessed using the QFT-Plus test and thus allowed us to systematically evaluate the utility of QFT-Plus quantitative IFNγ concentrations in predicting progression to symptomatic TB disease in multiple real-world, high-burden TB settings under the same clinical and laboratory protocols. Therefore, in this exploratory analysis, we evaluated baseline QFT-Plus quantitative TB1 and TB2 IFNγ concentrations across control, suspected TB, and lab-confirmed groups to assess their association and predictive performance for progression to symptomatic TB disease.

## METHODS

### Study Design

An epidemiological study to assess IGRA positivity was conducted from 20 December 2021 through 16 August 2024 (NCT05190146). Detailed study design, procedures, and results are described elsewhere [23]. Briefly, 7164 participants aged 15–34 years from high TB burden settings across 14 countries were enrolled and followed for a median of 17.1 months, with IGRA testing at baseline and Month 12 and continuous surveillance for suspected TB. Suspected TB cases (defined as participants presenting with unexplained cough, unexplained fever, night sweats, unintentional weight loss, or hemoptysis) prompted microbiological evaluation and additional blood sampling for HIV, IGRA, and exploratory biomarkers. Targeted preventive therapy (TPT) was not provided per study protocol but may have occurred under local standard of care. TPT use was rare, reported in <1% of participants prior to or at baseline [23].

### Laboratory Assessments

IGRA testing was conducted using the QFT-Plus assay (QuantiFERON®-TB Gold Plus, Qiagen, MD, USA) using modified manufacturer protocols for sample processing, specifically target goals for sample processing within 4 hours of sample collection and 20 hours incubation time for antigen stimulation [7]. This modified protocol was followed to reduce analytical variability [15–17]. QFT-Plus ELISA procedures followed the manufacturer's protocol and diagnostic algorithm.

For suspected TB visits, up to 3 sputum samples were collected preferably within 1 week, stored at 4°C, and shipped to the central laboratory within a target of 7 days. At the central laboratory, samples were decontaminated (1% final concentration NaOH) and processed. Samples were tested using the Xpert® MTB/RIF Ultra assay (Xpert Ultra, Cepheid, Sunnyvale, CA) per manufacturer instructions and inoculated into the BACTEC MGIT culture system (Becton Dickinson, Franklin Lakes, NJ, USA). MGIT cultures with a positive signal were confirmed as *Mtb* using MPT64 antigen testing. Contaminated MGIT cultures were decontaminated and reincubated. Final culture results were categorized as Negative, Contaminated (Inconclusive), Positive (MGIT+ MPT64+), or Positive (MGIT+ MPT64+) with Contamination.

### Exploratory Analysis Populations

Participants were included if they had a valid QFT-Plus result (ie, not indeterminate) obtained at central laboratory and all 4 baseline quantitative values available (NIL, TB1, TB2, and Mitogen) (n = 5246); participants from India were excluded due to unavailable quantitative data.

The control group is defined as participants who completed baseline and Month 12 visits and were not suspected of TB or diagnosed with TB during the study period.

The suspected TB group (n = 326) includes all participants who, during the study, developed signs or symptoms consistent with pulmonary TB but for whom protocol-specified microbiological testing did not confirm disease. For this analysis, participants with more than 2 sequential suspected TB visits within a 90-day period were excluded to reduce potential site-level heterogeneity in clinical evaluation practices, particularly at sites with frequent repeat suspected TB assessments in the absence of corresponding increases in lab-confirmed TB. The suspected TB group encompasses participants who triggered a suspected TB visit and had negative or contaminated laboratory results, as well as participants for whom study investigators initiated TB treatment despite the absence of microbiological confirmation (n = 31, classified as clinical TB). Quantitative IFNγ concentrations did not differ between participants categorized as clinical TB and those in the broader suspected TB group (data not shown); therefore, they were analyzed together in subsequent analyses as they together represented study participants with symptoms but no microbiological confirmation.

The lab-confirmed TB group (n = 23) is defined as participants with a microbiological confirmed pulmonary TB diagnosis within the study period in the per-protocol population. Lab-confirmed TB is defined in the protocol as having at least 1 positive *Mtb* MGIT culture and/or at least 1 positive Xpert Ultra assay, based on 3 sputum samples collected at 3 different visits preferably within 1 week. Within the lab-confirmed TB group, we further differentiated based on the concordance of different tests and samples. The stringent TB case group (n = 15) were defined as having 2 or more positive tests on 1 or more of the sputum samples collected during the suspected TB visit window. The single-positive TB case group (n = 8) were defined as only having a single-positive test (MGIT culture or Xpert Ultra) on only 1 of the sputum samples collected during the suspected TB visit window. For the Xpert Ultra assay, trace results were included in the positivity call in the TBV02-E01 primary analysis and thus were included in exploratory analyses as well (note: only 3 lab-confirmed TB cases in the exploratory population were determined using Xpert Ultra Trace results) [23]. Microbiological testing (eg, Xpert Ultra) was not performed at screening [23]. However, 22/23 lab-confirmed cases had no signs or symptoms of TB at baseline and were diagnosed >30 days after enrollment, consistent with an investigator-informed pragmatic definition of nonprevalent disease and aligned with recent published definitions [24]. One lab-confirmed case (single-assay positive case) was diagnosed shortly after enrollment.

### Statistical Analysis

Comparisons of quantitative IFNγ concentrations across TB outcome groups were performed using the Kruskal–Wallis test to evaluate global differences in medians. To elucidate which groups were responsible for a global difference, pairwise comparisons were performed using Dunn's test with Bonferroni correction; adjusted *P* values (q values) are reported and used for interpretation (Supplementary Table 1). Spearman's rank correlation coefficient (Spearman's ρ) was used to assess associations between baseline quantitative IGRA values and the number of days to lab-confirmed TB diagnosis. All statistical analyses were conducted using the SciPy library in Python (version 1·15·2; https://scipy.org/).

TB1, TB2, and the difference between antigen tube IFNγ concentrations (TB2-TB1), were first compared across TB outcome groups in the full exploratory population as all groups had participants who were IGRA positive or negative at baseline (Table 1). The QFT-Plus test qualitative output is determined by the manufacturer's algorithm using NIL, TB1, TB2, and mitogen values, with positivity defined as a NIL-subtracted TB1 or TB2 value that is ≥0.35 International Units per milliliter (IU/mL) and >25% of the NIL value [7]. By design, IGRA-negative individuals generally have low TB1 and TB2 IFNγ concentrations, potentially biasing analyses. To address this, we also completed an analysis restricted to IGRA-positive participants at baseline.

**Table 1.** Baseline Characteristics of the Exploratory Analysis Population

		Control	Suspected TB	Lab-Confirmed TB	
				Single-Positive Sputum Test	Stringent (≥2 Positive Sputum Tests)
N		4897	326	8	15
Age (mean and SD)		24.2 (4.9)	24.7 (4.9)	20.8 (4.7)	27 (4.1)*
Male		46.3%	52.5%	25.0%	53.3%
Race	Black	55.3%	77.3%	87.5%	46.7%
	Asian	37.0%	15.2%	12.5%	46.7%
	White	2.1%	3.7%	0.0%	0.0%
	Multiple	5.4%	4.0%	0.0%	0.0%
	Other	0.2%	0.0%	0.0%	6.7%
Healthcare worker/healthcare student		3.7%	0.9%	0.0%	0.0%
Body mass index (BMI) (m/kg^2^) (mean and SD)		24 (5.6)	23.4 (6.3)	25.4 (9.0)	21.3 (5.0)
Known BCG vaccination		79.2%	50.3%	50.0%	66.7%
HIV positive		4.7%	10.1%	12.5%	20.0%**
IGRA+ at baseline		31.1%	40.5%	12.5%	86.7%***
Follow-up duration, days (mean [SD])		533 (86)	592 (101)	534 (179)	525 (135)
Time to TB diagnosis, days (mean [SD])		Not applicable	Not applicable	320 (239)	236 (133)

Demographics of comparator groups in the exploratory analysis population. The lab-confirmed population is divided into 2 subsets, single-positive case definition (single-positive) and stringent case definition (stringent). Means and standard deviations (SD) or percentage of exploratory analysis population is shown.

Pairwise comparisons between control and stringent populations were performed for all baseline characteristics; statistically significant differences are indicated: *P = .02, **P = .03, ***P < .0001.

### Evaluation of Predictive Value

The utility of quantitative IGRA values as predictive biomarkers for symptomatic TB disease development was evaluated using sensitivity, specificity, and positive predictive values (PPVs), which were calculated based on the ability to differentiate between pairs of TB outcome groups over the study follow-up period (listed in Table 1). Additionally, Youden's J, F1-score, and AUROC were calculated. ROC curves, PPV, and F1-score were calculated using scikit-learn in python (version 1·6·1; https://scikit-learn.org/).

### Role of the Funding Source

The funder of this study had no role in study design, study execution, or writing of the report.

## RESULTS

This exploratory analysis included 5246 participants from 13 countries: 4897 in the control group, 326 in the suspected TB group, and 23 in the lab-confirmed TB group (15 meeting stringent case definitions and 8 meeting single-positive case definitions) (Table 1). Overall population characteristics were similar between the full study [23] and this exploratory analysis subset. Baseline characteristics were generally comparable across groups; however, participants meeting the stringent case definition were significantly older and had a higher proportion of people living with HIV and baseline IGRA positivity compared with controls (Table 1). The mean follow-up duration in the study was comparable across groups (525–592 days, Table 1).

**Figure 1.**
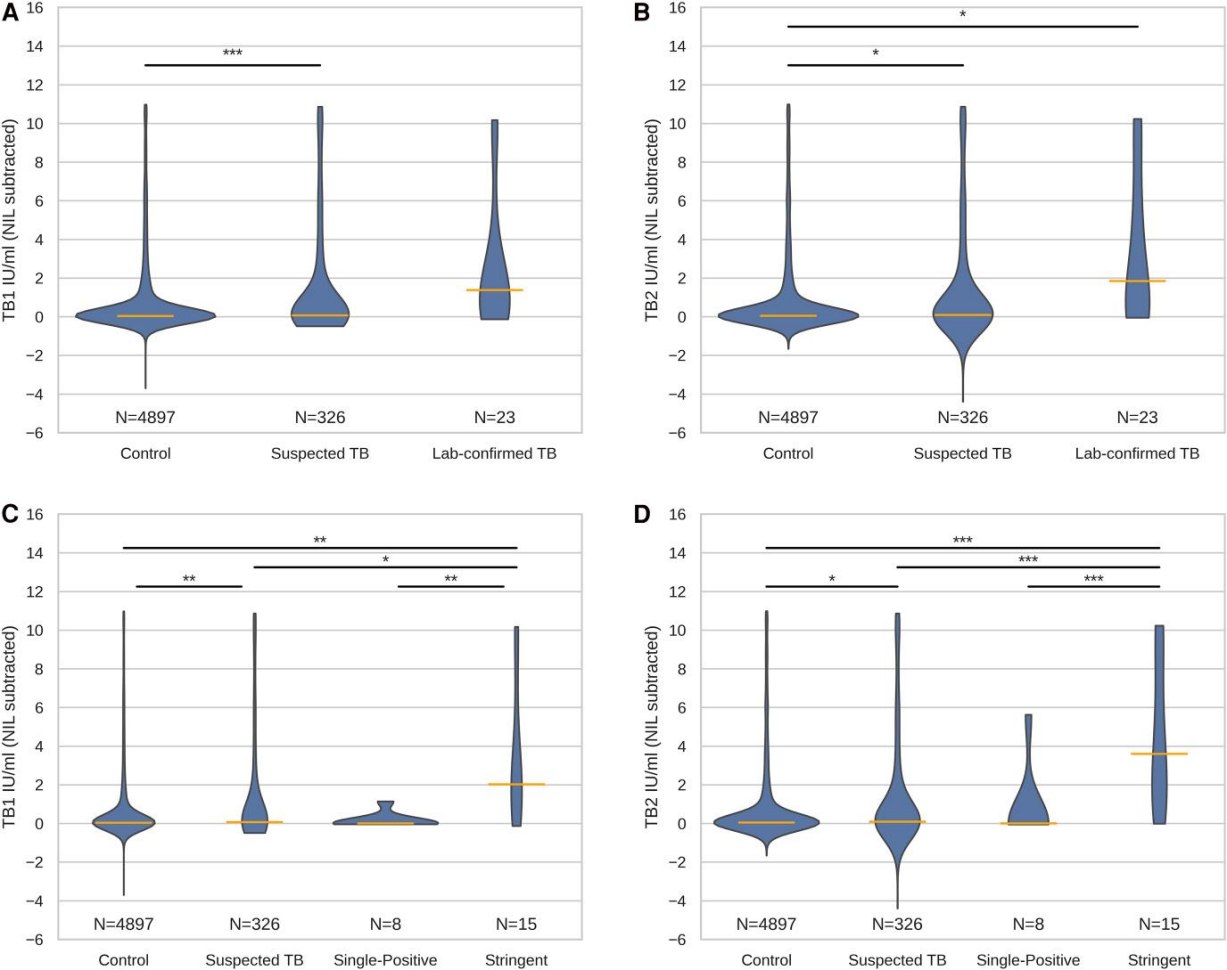
Baseline TB1 and TB2 IFNγ concentrations among participants by TB outcome classification in IGRA-positive and -negative participants. (*A* and *B*) Violin plots depict IFNγ concentrations (IU/mL, NIL-subtracted) from TB1 (*A*) and TB2 (*B*), antigen tubes measured at baseline among participants who did not develop TB (Control), were suspected of TB but not microbiologically confirmed (suspected TB), or had lab-confirmed TB during follow-up. Statistical comparison by Kruskal–Wallis test showed significant differences across groups for all measures: TB1 (*P* = .0001), TB2 (*P* = .0003). (*C* and *D*) Box plots show baseline TB1 (*C*) and TB2 (*D*) IFNγ levels stratified by more granular TB outcome groups: control, suspected TB, single-assay positive TB cases (1 positive culture or Xpert result), and Stringent TB Cases (≥2 positive microbiological results and/or sputum samples). Kruskal–Wallis tests were significant for TB1 (*P* = .000002), TB2 (*P* = .0000008). Pairwise comparisons *P < .05, **P < .01, ***P < .001. Orange bar represents median value per group.

Using the full exploratory cohort, we analyzed the difference in baseline IFNγ concentrations between participants who did not progress to TB (controls) compared with participants who were later suspected of TB but with no laboratory confirmation and participants who later developed TB with lab confirmation. We intentionally included both IGRA-positive and IGRA-negative participants at baseline to allow us to evaluate QFT-Plus quantitative values as a continuous measure and assess their potential role as a one-time screening or stratification biomarker for clinical research applications in broad populations. At baseline, TB1 IFNγ concentrations were significantly higher in the suspected TB group compared with the control group (0.07 vs 0.03 IU/mL, *P* = .0009) with a trend toward higher IFNγ levels in the lab-confirmed TB group compared with the control (1.38 vs 0.03 IU/mL, *P* = .09) (Figure 1*A*). For TB2, IFNγ levels were significantly higher in both suspected and lab-confirmed TB groups relative to the control group (0.09 vs 0.04 IU/mL, *P* = .01; 1.84 vs 0.04 IU/mL, *P* = .01, respectively) (Figure 1*B*).

We next compared baseline IFNγ concentrations between groups in the full exploratory cohort using stringent and single-positive TB case definitions. The stringent case definition mirrors the primary endpoint being adopted in current and upcoming TB vaccine efficacy trials [25] and therefore provides a relevant benchmark for evaluating endpoint-matched biomarker performance. Compared with the control group, baseline TB1 and TB2 IFNγ concentrations were significantly higher in participants with suspected TB (TB1: 0.07 vs 0.03 IU/mL, *P* = .001; TB2: 0.09 vs 0.04 IU/mL, *P* = .02) and those meeting the stringent case definition (TB1: 2.02 vs 0.03 IU/mL, *P* = .001; TB2: 3.59 vs 0.04 IU/mL, *P* = .00003), but not in participants meeting single-positive case definition (Supplementary Table 1; Figure 1*C* and *D*). Participants meeting the stringent case definition had significantly higher TB1 and TB2 IFNγ concentrations than those meeting the single-positive case definition (Supplementary Table 1; Figure 1*C* and *D*). Notably, median TB2 IFNγ concentration in participants meeting the stringent case definition (3.59 IU/mL, Figure 1*D*) was markedly higher when compared with the median of the broader lab-confirmed group (1.84 IU/mL, Figure 1*B*).

**Figure 2.**
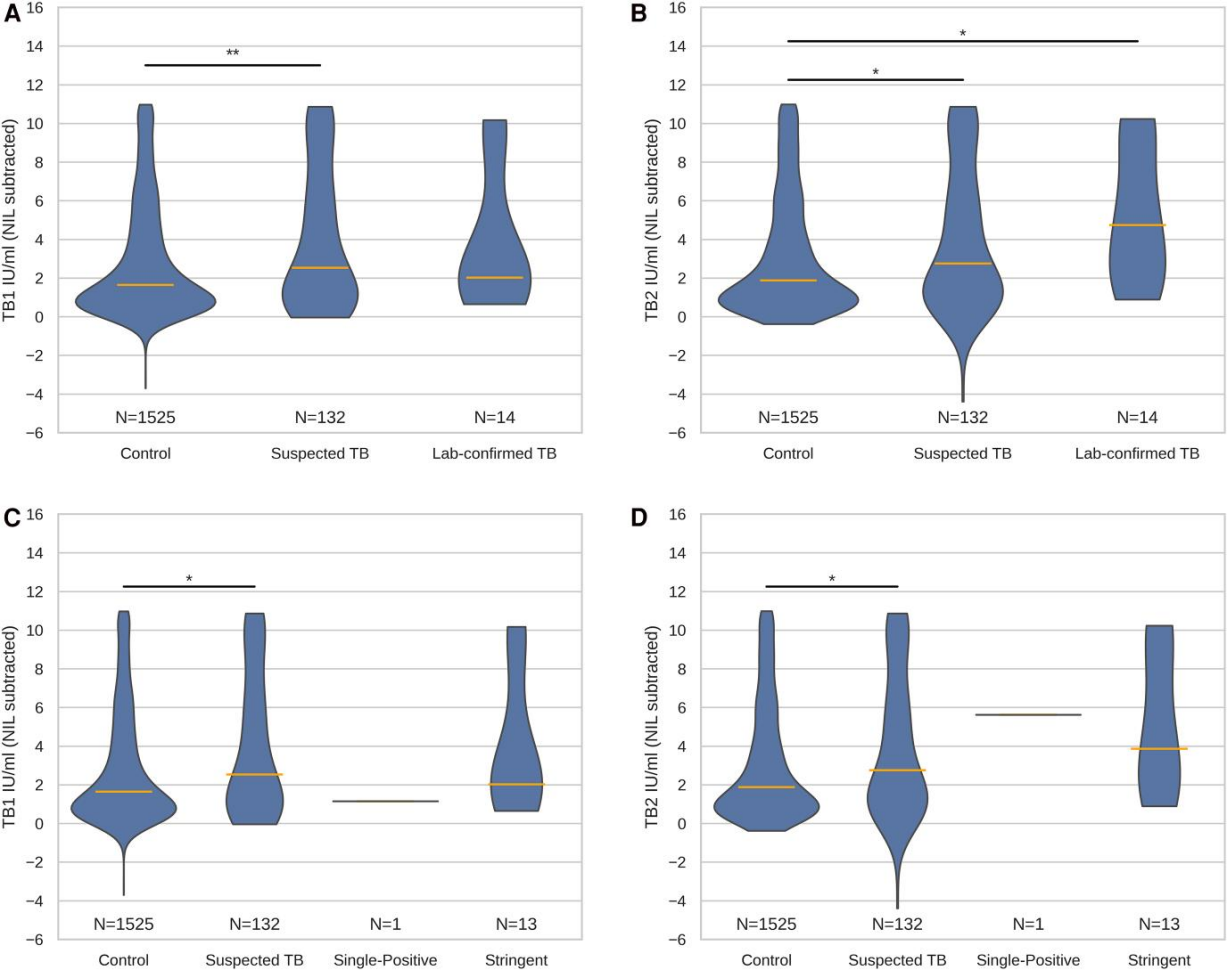
Baseline TB1 and TB2 IFNγ concentrations by TB outcome classification in IGRA-positive participants. (*A* and *B*) Violin plots show IFNγ concentrations (IU/mL, NIL-subtracted) from TB1 (*A*) and TB2 (*B*) antigen tubes measured at baseline among IGRA-positive participants who did not develop TB (Control), were suspected of TB but not microbiologically confirmed (Suspected TB), or had lab-confirmed TB during follow-up. Kruskal–Wallis tests showed significant group differences for TB1 (*P* = .002), TB2 (*P* = .001). (*C* and *D*) Box plots display TB1 (*C*) and TB2 (*D*) IFNγ concentrations stratified by TB outcome groups: Control, Suspected TB, Single-assay positive TB cases (1 positive culture or Xpert result), and Stringent TB cases (≥2 positive microbiological results and/or sputum samples). Kruskal–Wallis tests showed significant group differences for TB1 (*P* = .006), TB2 (*P* = .003). Pairwise comparisons *P < .05, **P < .01, ***P < .001. Orange bar represents median value per group.

We then restricted the analysis to participants who were IGRA-positive at baseline to reduce bias from low IFNγ values in IGRA-negative individuals and to assess the biomarker's use in a population already at elevated risk of progression, specifically its ability to further stratify risk. This reduced our TB cases as only 14/23 lab-confirmed participants were IGRA positive at baseline (n = 13 stringent case definition, n = 1 single-positive). In IGRA-positive participants, TB1 IFNγ concentrations were significantly higher in the suspected TB group compared with control (2.5 vs 1.64 IU/mL, *P* = .006), but not in the lab-confirmed TB group (2.02 vs 1.64 IU/mL, *P* = .3) (Figure 2*A*) or in participants meeting stringent case definition (2.02 vs 1.64 IU/mL, *P* = .4) (Figure 2*C*). For TB2, IFNγ concentrations were significantly higher in both the suspected TB and lab-confirmed TB groups relative to control group (2.75 vs 1.88 IU/mL, *P* = .01; 4.74 vs 1.88 IU/mL, *P* = .02) (Figure 2*B*), with higher levels in the smaller number of IGRA-positive participants meeting the stringent case definition although the difference was not significant (3.86 vs 1.88 IU/mL, *P* = .09) (Figure 2*D*).

Given recent findings that the TB2-TB1 IFNγ difference is elevated in active or recent TB infection [21, 22], we repeated all analyses using the TB2-TB1 metric but found no baseline differences between controls and lab-confirmed or stringent cases (Supplementary Table 1), suggesting this measure may better reflect active disease rather than used to predict progression, consistent with prior reports [13].

**Figure 3.**
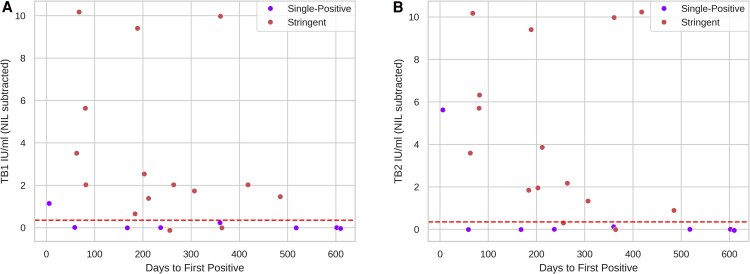
Correlation between Baseline IFNγ concentrations and time to TB diagnosis among Lab-confirmed TB cases. Scatter plots show baseline IFN-γ concentrations (NIL-subtracted, in IU/mL) for (*A*) TB1 and (*B*) TB2 plotted against the number of days from baseline to first positive TB laboratory test. Spearman correlation coefficients (ρ) and *P* values for (*A*) ρ = −0.36, *P* = .08 and (*B*) ρ = −0.34, *P* = .10. Dashed red lines indicate the manufacturer's recommended positivity threshold of 0.35 IU/mL. Stringent case definition (red), single-positive case definition (purple).

Analysis of correlation between IFNγ concentrations at baseline and time to diagnosis of lab-confirmed TB disease showed trends between baseline TB1 or TB2 IFNγ concentration and days to first positive laboratory test (TB1: r = −0.36, *P* = .08; TB2: r = −0.34, *P* = .10; Figure 3).

**Figure 4.**
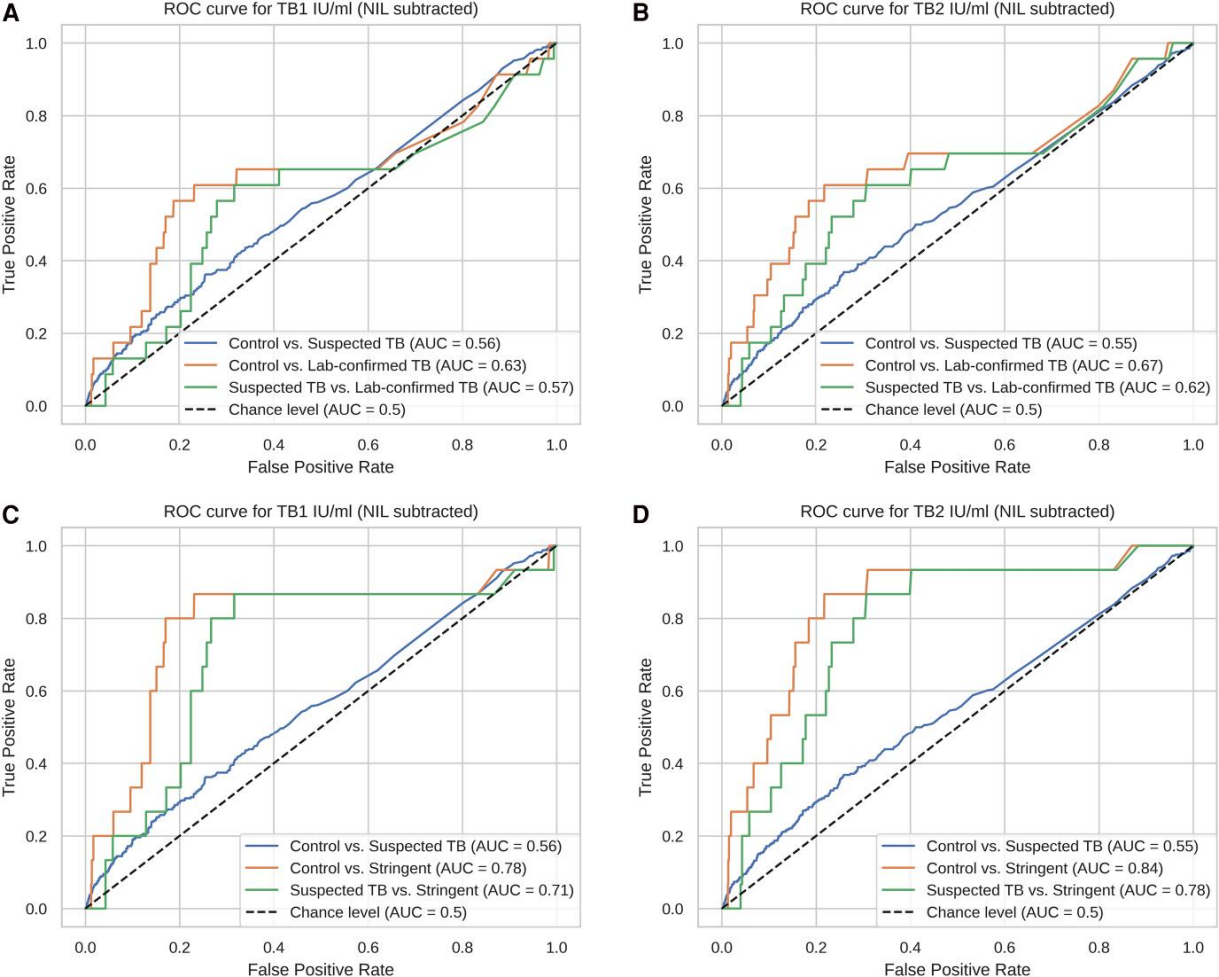
Receiver operating characteristic (ROC) curve analyses of baseline IFNγ concentrations for predicting TB disease progression. (*A* and *B*) depict ROC curves for distinguishing outcome groups using baseline IFNγ levels from (*A*) TB1 and (*B*) TB2 in the analysis based on lab-confirmed TB cases (n = 23). Comparisons include Control versus Suspected TB (blue), Control versus Lab-Confirmed TB (orange), and Suspected TB versus Lab-Confirmed TB (green). (*C* and *D*) show ROC curves for the same IFNγ measures, (C) TB1 and (D) TB2 in the stringent case definition subgroup (n = 15). Comparisons include control versus suspected TB (blue), Control versus Stringent (orange), and Suspected TB versus Stringent (green). Area under the curve (AUC) values are indicated for each comparison. Diagonal dashed lines indicate the line of no discrimination (AUROC = 0.5).

Building on the observed associations between baseline IFNγ concentrations and subsequent TB outcomes, we next evaluated the sensitivity, specificity, and PPV of TB1 and TB2 measures. Given our interest in assessing the value of quantitative IFNγ measures as a single baseline biomarker for clinical research applications, including trial enrollment and stratification, we evaluated TB1 and TB2 IFNγ concentrations as a one-time measure independent of baseline IGRA status to predict TB development over the follow-up period. When differentiating laboratory-confirmed TB cases from controls, both markers showed similar performance in sensitivity (TB1: 52%; TB2: 57%) and specificity (TB1: 81%; TB2: 78%; (Table 2). Area under the receiver operating characteristic curve (AUROC) values were also comparable across markers (TB1: 0.63; TB2: 0.67) (Figure 4*A* and *B*).

**Table 2.** Predictive Performance of Baseline IFNγ Concentrations from QFT-Plus (TB1, TB2) for Progression to TB

Lab-confirmed						
	Group 1	Group 2	Youden Cutoff	Sensitivity	Specificity	PPV
TB1	Control	Suspected TB	0.50	36%	75%	8.6%
	Control	Lab-confirmed TB	1.14	52%	81%	1.3%
	Suspected TB	Lab-confirmed TB	0.65	57%	68%	11.1%
TB2	Control	Suspected TB	0.55	37%	74%	8.6%
	Control	Lab-confirmed TB	0.89	57%	78%	1.2%
	Suspected TB	Lab-confirmed TB	0.89	57%	70%	11.6%

Stringent case definition						
	Group 1	Group 2	Youden cutoff	Sensitivity	Specificity	PPV
TB1	Control	Suspected TB	0.5	36%	75%	8.6%
	Control	Stringent case	0.65	80%	77%	1.0%
	Suspected TB	Stringent case	0.65	80%	68%	10.3%
TB2	Control	Suspected TB	0.55	37%	74%	8.7%
	Control	Stringent case	0.89	80%	78%	1.1%
	Suspected TB	Stringent case	0.89	80%	70%	10.7%

Sensitivity, specificity, positive predictive value (PPV), and Youden's J-derived optimal cutoff values are presented for each IFNγ measure across comparator groups. Analyses were conducted for 2 case definitions: (1) Lab-confirmed TB (n = 23) versus controls (n = 4897) and suspected TB (n = 326), and (2) Stringent case definition (n = 15) versus controls and suspected TB. The Youden Cutoff represents the IFNγ threshold (in IU/mL) at which sensitivity and specificity are optimized.

Performance improved further when applying the more stringent case definition and distinguishing against controls, with similar sensitivity (TB1: 80%; TB2: 80%) and specificity (TB1: 77%, TB2: 78%), exceeding the minimal WHO-recommended test thresholds for a predictive test for progression to TB disease [4]. AUROC values were highest for TB2 (0.84) (Figure 4*D*). The PPV for progression to TB disease within 2 years was low across all measures (Table 2). Youden's J values for distinguishing stringent TB cases from controls were higher than the standard QFT-Plus diagnostic cutoff of ≥0.35 IU/mL, with values of 0.65 IU/mL (TB1) and 0.89 IU/mL (TB2), suggesting that more strict cutoffs outside the diagnostic uncertainty zone [16] may also improve predictive accuracy to symptomatic disease.

## DISCUSSION

We observed that higher baseline TB2 IFNγ concentrations were associated with subsequent progression to laboratory-confirmed pulmonary TB disease, with the strongest elevations observed in participants meeting the stringent case definition. Association of baseline TB1 IFNγ concentration and eventual symptomatic disease progression was found only when including both including IGRA-positive and −negative individuals and in participants classified under a stringent TB case definition. However, consistent with previous studies [12–14], we found substantial variability and considerable overlap in IFNγ concentrations between participants who did and did not progress to symptomatic TB disease as well as no significant association between baseline measurements and time to laboratory confirmed TB. In addition, the relatively limited number of TB cases negatively impacted the observed PPV of these measures. Although promising, these observations highlight the current limitations of using QFT-Plus quantitative IFNγ measurements alone as reliable predictive biomarkers to symptomatic TB disease.

When considering the dynamic TB spectrum, increasing TB-specific IFNγ concentrations may reflect evolving host-*Mtb* interactions, with rising mycobacterial burden resulting in increased TB-specific T-cell activation and effector responses [8, 26–28]. The elevated IFNγ responses observed in this state could signal early breakdown of immune containment, preceding progression to symptomatic disease. As no direct measurement of the presence of TB (ie, Xpert Ultra) was performed at baseline in this study, we were unable to unequivocally identify or exclude asymptomatic TB among participants in this analysis. Future studies specifically incorporating populations with and preceding asymptomatic TB [29] are needed to determine how TB1 and TB2 IFNγ concentrations perform across the full TB disease spectrum. For clinical or research applications in which distinguishing asymptomatic TB from other disease states is not required, QFT-Plus quantitative measures may still provide added value.

In this study, baseline TB2 IFNγ concentrations were progressively higher among participants in the suspected TB group (without microbiological confirmation), the lab-confirmed TB group (at least 1 positive microbiological test), and those meeting a stringent case definition (at least 2 positive microbiological tests). This gradient suggests that the strength of association between TB2 IFNγ concentrations and TB outcomes increases with greater diagnostic certainty. Only when including participants meeting the stringent case definition did both TB1 and TB2 IFNγ measures exceed the WHO minimum test characteristics (>75% sensitivity, >75% specificity) for predicting progression to symptomatic TB [4]. These findings have practical implications for clinical research settings where a biomarker may be needed as a single baseline screening tool to enrich or stratify participants in prevention studies. Notably, recent TB vaccine trials have adopted stringent case definitions as their primary efficacy endpoint. Under these conditions, quantitative IFNγ concentrations, particularly TB2 values, may offer meaningful predictive or stratification utility beyond the binary classification afforded by qualitative QFT-Plus result. These findings also highlight that with continuous efforts to refine endpoint definitions for TB vaccine and therapeutics trials [30], candidate biomarkers should be re-evaluated to ensure that their performance aligns with the specificity of the selected endpoints, thereby minimizing the risk of dismissing potentially valuable markers that underperform under broader case definitions.

This exploratory analysis has several limitations. As an exploratory analysis of data from a large epidemiological study, it was not specifically powered to assess the predictive performance of quantitative IFNγ concentrations from the QFT-Plus assay. A key limitation is the small number of TB cases, which limits statistical power and precluded robust subgroup analyses that would help determine the generalizability and stability of these associations across subsets. However, the data generated provides an important foundation for building power calculations to inform the design of future studies which can rigorously assess the utility of these measures. This study was limited to pulmonary TB cases in participants aged 15–34 years, and the findings may not be generalizable to extrapulmonary TB or other age groups.

Finally, this analysis interprets baseline QFT-Plus measurements as reflective of future TB disease risk; however, participants were enrolled in high-transmission settings with ongoing exposure risk. As such, some TB cases, particularly those occurring later during follow-up, may represent new infections acquired after baseline rather than progression from pre-existing infection. Notably, 2 participants who were IGRA-negative at baseline subsequently converted and developed active TB during follow-up, supporting the contribution of incident infection. We were unable to account for or quantify differences in exposure risk across sites, which may have influenced both baseline IGRA responses and subsequent disease risk. These factors limit the ability to attribute progression risk solely to baseline QFT-Plus responses. However, baseline IFNγ concentrations may still capture an underlying state of heightened susceptibility, in which subsequent exposures further amplify progression risk, supporting their potential utility for risk stratification even in high-transmission settings. This limitation is not unique to our study but reflects a broader challenge in the field. Distinguishing risk captured at baseline from ongoing or incident infection remains an important area for future study and is critical for interpreting baseline biomarkers and optimizing risk stratification strategies.

In conclusion, QFT-Plus IFNγ concentrations, and TB2 values in particular, may be informative measures as early indicators of symptomatic TB disease progression. While limited in standalone predictive value, the associations between these biomarkers in individuals who progress to symptomatic TB disease as defined under the stringent TB case definition are promising for future clinical research applications.

## GATES MRI TB EPI STUDY GROUP

Q. Bhorat (Soweto Clinical Trials Centre, Johannesburg), M. Loveday (Botha's Hill CRS, Durban, South Africa), C. Duran Palma (Trident Clinical, Homestead, South Africa), J. Lombaard (Josha Research, Mangaung, South Africa), S. Cossie (CRISMO Bertha Gxowa Research Centre, Ekurhuleni, Gauteng, South Africa), J.G. Geldenhuys (FCRN Clinical Trial Centre Pty Ltd, Three Rivers Vereeniging, Gauteng, South Africa), K. Ahmed (Setshaba Research Centre, Soshanguve, Gauteng, South Africa), E. Spooner (PHOENIX Pharma (Pty) Ltd, HIV Prevention Research Unit, Ethekwini, Kwazulu—Natal, South Africa), L. Tina (Victoria Biomedical Research Institute, US Army Medical Research Unit, Kombewa Clinical Research Centre, Kisumu, Western, South Africa), B. Ogutu (Strathmore University, Centre for Clinical Research, Kisumu, Western, South Africa), R. McClelland (Ganjoni Municipal Communicable Diseases Control Centre, Mombasa, Kenya), W. Kilembe (Center for Family Health Research in Zambia (CFHRZ)—Lusaka, Zambia Emory HIV Research Project Lusaka, Lusaka, Zambia), S. Kwame (Zambart University of Zambia, Zambart, RidgewayCampus, Lusaka, Zambia), M. Inambao (Center for Family Health Research in Zambia (CFHRZ)—Ndola, Northrise, Zambia), E. Sprinz (Hospital de Clinicas de Porto Alegre (HCPA)—PPDS, Floresta Porto Alegre—RS, Porto Alegre, Rio Grande do Sul, Brazil), E. Cadena (Philippine Tuberculosis Society Inc, Quezon City, National Capital Region, Philippines), J.R. Gonong (Lung Center of The Philippines, Quezon City, Philippines), E.J. Berame (Healthlink Iloilo, Iloilo City, Iloilo, Philippines), M.G.D. Isidro (West Visayas State University Medical Center, Iloilo City, Iloilo, Philippines), G. Zabat (Health Cube Medical Clinics, Quezon City, Philippines), R.G.M. Veto (Tropical Disease Foundation, Makati City, Philippines), Javier R. Lama (Asociación Civil Impacta Salud y Educación, Lima, Peru), E. Sanchez (Hospital Nacional Sergio E. Bernales, Lima, Peru), V. Nankabirwa (Makerere University—School of Public Health, Kampala, Uganda), H.L. Nguyen (Pham Ngoc Thach Hospital, Phường Số 12 Quận 5, Vietnam), V.N. Nguyen (National Lung Hospital, Hanoi, Vietnam), G. Marks (Woolcock Institute Of Medical Research, Hanoi, Vietnam), B. Alisjahbana (Universitas Padjadjaran, Bandung, Jawa Barat, Indonesia), Burhan E. (Persahabatan Hospital, Jakarta, Indonesia), Kaswandani N. (Puskesmas Kecamatan Kramat Jati, Jakarta, Indonesia), G. Luyeye Matondo Mandiangu (Centre de Sante Maternite Binza, Ngaliema, Kinshasa, Democratic Republic of the Congo), H.Z. Swah Koko (Centre de Sante 2eme Rue, Limete, Kinshasa, Democratic Republic of the Congo), E. Mwamba Kabunda (Hopital General de reference de Kabinda, Lingwala, Kinshasa, Democratic Republic of the Congo), S. Viegas (Instituto Nacional de Saude, Marracuene, Mozambique), and A.L. Garcia-Basteiro (Centro de Investigação em Saúde de Manhiça (CISM), Maputo, Mozambique).

## Supplementary Material

ofag437_Supplementary_Data

## Supplementary Data

Supplementary materials are available at *Open Forum Infectious Diseases* online. Consisting of data provided by the authors to benefit the reader, the posted materials are not copyedited and are the sole responsibility of the authors, so questions or comments should be addressed to the corresponding author.

## Notes

***Author contributions.*** J.S. and M.S. were involved in strategy and data analysis. A.F.D. and A.C.S. were involved in the conception or design of the trial. J.S., M.S., A.F.D., L.L.H., A.C., D.G., A.A.H., M.T.G., S.R.H., W.A.H., J.R.L., M.M., S.M., V.N., V.C.R., T.R., J.S., C.K., A.W., and T.M.W. contributed to data collection or data generation. J.S., M.S., A.F.D., A.C.S., A.C., and N.F. contributed to data interpretation. M.S. accessed and verified the data. J.S. and N.F. were responsible for the decision to submit the manuscript. All authors reviewed the draft critically, approved the final version to be submitted, and took accountability for all aspects of the published work.

***Acknowledgements.*** We are grateful to the study participants and their families for taking part in this trial and all clinical investigators, independent ethics committee members, site staff, PPD staff, and BARC staff for their contributions. We would like to thank all members of the TBV02-E01 Study Group including clinical operations. The authors thank Douglas Wilson for analysis support. This study was funded by the Gates Foundation [INV-008522]. The conclusions and opinions expressed in this work are those of the authors alone and shall not be attributed to the Foundation. Under the grant conditions of the Foundation, a Creative Commons Attribution 4.0 License has already been assigned to the Author Accepted Manuscript version that might arise from this submission. Medical writing assistance was provided by Madeeha Aqil, PhD, CMPP^TM^ and was funded by the Gates Medical Research Institute.

***Data availability.*** Anonymized participant-level data may be shared with external researchers in accordance with the study participants' written and executed informed consent document and any local or applicable regulations on data sharing. Qualified researchers may submit a request for anonymized participant-level data along with a research proposal to Gates MRI's Data Protection Officer at dataprotection-officer@gatesmri.org for review. The types of supporting information that could be shared with external researchers include: the Study Protocol, Statistical Analysis Plan, Informed Consent Form, Clinical Study Report, and analytic code. A data sharing agreement must be in place before any study data are shared. There are additional circumstances that may prevent the sharing of data with external researchers, including but not limited to contractual obligations to existing partners and any restrictions imposed by regulatory bodies.

***Potential conflicts of interests.*** J.S., M.S., A.F.D., L.L.H., D.G., A.A.H., M.T.G., S.R.H., W.A.H., J.R.L., M.M., S.M., V.N., T.R., J.S., C.K., A.W., T.M.W., A.C.S., A.C., and N.F. declare no competing interests. M.T.G. reports funds from Gates MRI and PPD for implementing study activities. V.C.R. reports support for TBV02-E01 study activities from Brazil's Departamento de Ciência e Tecnologia (Secretaria de Ciência e Tecnologia, Ministério da Saúde) and NIH/NIAID grants, research funds from TB Alliance for Drug Development, Impacto da COVID-19 Nas Manifestações Clínicas, Diagnóstico, Desfecho Do Tratamento e Resposta Imune para Tuberculose Pulmonar, Associative BRICS Research in COVID-19 and Tuberculosis (ABRICOT), RePORT International Data Harmonization, Transcriptional Signature of TB in advanced HIV, Predictors of Resistance Emergence Evaluation in Multidrug-resistant Tuberculosis patients on Treatment—PREEMPT, Caribbean, Central, and South America network for HIV Epidemiology (CCASAnet), Immunogenetic predictors of active and incipient TB in HIV-negative and -positive close TB contacts, consulting fees from ONU-OMS for an HIV response review in Myanmar, honoraria for lectures from GlaxoSmithKline Brasil Ltda, Qiagen, and Virology education events, travel support from ViiV 18 European AIDS conference. All other authors report no potential conflicts.
