# Supplementary material for: Associations Between QuantiFERON-TB Gold Plus IFNγ Concentrations and Progression to Symptomatic Tuberculosis in Global High-Burden TB Settings
Source: Open Forum Infect Dis. 2026 Jul 24;13(7):ofag437. doi: 10.1093/ofid/ofag437 (PMC13397382; doi:10.1093/ofid/ofag437)
Supplement: ofag437_Supplementary_Data [file ofag437_supplementary_data.pdf]

Analyte	Group 1	Group 1 N	Group 1 median	Group 1 IQR	Group 2	Group 2 N	Group 2 median	Group 2 IQR	Median difference	p-value	q-value
Full cohort (IGRA+ and IGRA-)											
TB1 IU/ml (NIL subtracted)	Control	4897	0.03	0.53	Suspected TB	326	0.07	1.6975	0.04	0.000301	0.000902
TB1 IU/ml (NIL subtracted)	Control	4897	0.03	0.53	Lab-confirmed TB	23	1.38	2.275	1.35	0.030592	0.091777
TB1 IU/ml (NIL subtracted)	Suspected TB	326	0.07	1.6975	Lab-confirmed TB	23	1.38	2.275	1.31	0.255793	0.76738
TB2 IU/ml (NIL subtracted)	Control	4897	0.04	0.62	Suspected TB	326	0.09	1.68	0.05	0.003738	0.011214
TB2 IU/ml (NIL subtracted)	Control	4897	0.04	0.62	Lab-confirmed TB	23	1.84	5.66	1.8	0.005454	0.016362
TB2 IU/ml (NIL subtracted)	Suspected TB	326	0.09	1.68	Lab-confirmed TB	23	1.84	5.66	1.75	0.054433	0.1633
TB2 minus TB1	Control	4897	0	0.06	Suspected TB	326	0	0.05	0	0.01456	0.043679
TB2 minus TB1	Suspected TB	326	0	0.05	Lab-confirmed TB	23	0	0.295	0	0.089176	0.267529
TB2 minus TB1	Control	4897	0	0.06	Lab-confirmed TB	23	0	0.295	0	0.277498	0.832494
TB1 IU/ml (NIL subtracted)	Control	4897	0.03	0.53	Stringent	15	2.02	3.15	1.99	0.000169	0.001012
TB1 IU/ml (NIL subtracted)	Control	4897	0.03	0.53	Suspected TB	326	0.07	1.6975	0.04	0.000301	0.001803
TB1 IU/ml (NIL subtracted)	Single-Positive	8	0	0.075	Stringent	15	2.02	3.15	2.02	0.000626	0.003754
TB1 IU/ml (NIL subtracted)	Suspected TB	326	0.07	1.6975	Stringent	15	2.02	3.15	1.95	0.003723	0.022339
TB1 IU/ml (NIL subtracted)	Suspected TB	326	0.07	1.6975	Single-Positive	8	0	0.075	-0.07	0.04098	0.245882
TB1 IU/ml (NIL subtracted)	Control	4897	0.03	0.53	Single-Positive	8	0	0.075	-0.03	0.138175	0.82905
TB2 IU/ml (NIL subtracted)	Control	4897	0.04	0.62	Stringent	15	3.59	6.275	3.55	5.28E-06	3.17E-05
TB2 IU/ml (NIL subtracted)	Single-Positive	8	0	0.04	Stringent	15	3.59	6.275	3.59	8.91E-05	0.000535
TB2 IU/ml (NIL subtracted)	Suspected TB	326	0.09	1.68	Stringent	15	3.59	6.275	3.5	0.000128	0.000766
TB2 IU/ml (NIL subtracted)	Control	4897	0.04	0.62	Suspected TB	326	0.09	1.68	0.05	0.003738	0.022428
TB2 IU/ml (NIL subtracted)	Suspected TB	326	0.09	1.68	Single-Positive	8	0	0.04	-0.09	0.04921	0.295263
TB2 IU/ml (NIL subtracted)	Control	4897	0.04	0.62	Single-Positive	8	0	0.04	-0.04	0.128404	0.770424
TB2 minus TB1	Control	4897	0	0.06	Suspected TB	326	0	0.05	0	0.01456	0.087357
TB2 minus TB1	Suspected TB	326	0	0.05	Stringent	15	0.07	0.81	0.07	0.032632	0.195793
TB2 minus TB1	Control	4897	0	0.06	Stringent	15	0.07	0.81	0.07	0.100706	0.604239
TB2 minus TB1	Control	4897	0	0.06	Single-Positive	8	0	0.015	0	0.166667	1
TB2 minus TB1	Suspected TB	326	0	0.05	Single-Positive	8	0	0.015	0	0.166667	1
TB2 minus TB1	Single-Positive	8	0	0.015	Stringent	15	0.07	0.81	0.07	0.166667	1
IGRA+ Populations Only											
TB1 IU/ml (NIL subtracted)	Control	1525	1.64	3.83	Suspected TB	132	2.525	5.5725	0.885	0.002105	0.006314
TB1 IU/ml (NIL subtracted)	Control	1525	1.64	3.83	Lab-confirmed TB	14	2.02	3.5725	0.38	0.112177	0.33653
TB1 IU/ml (NIL subtracted)	Suspected TB	132	2.525	5.5725	Lab-confirmed TB	14	2.02	3.5725	-0.505	0.333333	1
TB2 IU/ml (NIL subtracted)	Control	1525	1.88	4.17	Suspected TB	132	2.755	5.4075	0.875	0.006092	0.018277
TB2 IU/ml (NIL subtracted)	Control	1525	1.88	4.17	Lab-confirmed TB	14	4.74	6.625	2.86	0.009901	0.029703
TB2 IU/ml (NIL subtracted)	Suspected TB	132	2.755	5.4075	Lab-confirmed TB	14	4.74	6.625	1.985	0.114476	0.343428
TB2 minus TB1	Control	1525	0.03	0.56	Suspected TB	132	0	0.4125	-0.03	0.020679	0.062038
TB2 minus TB1	Suspected TB	132	0	0.4125	Lab-confirmed TB	14	0.075	2.1575	0.075	0.087893	0.26368
TB2 minus TB1	Control	1525	0.03	0.56	Lab-confirmed TB	14	0.075	2.1575	0.045	0.315007	0.945021
TB1 IU/ml (NIL subtracted)	Control	1525	1.64	3.83	Suspected TB	132	2.525	5.5725	0.885	0.002105	0.012627
TB1 IU/ml (NIL subtracted)	Control	1525	1.64	3.83	Stringent	13	2.02	3.9	0.38	0.080648	0.483887
TB1 IU/ml (NIL subtracted)	Control	1525	1.64	3.83	Single-Positive	1	1.14	0	-0.5	0.166667	1
TB1 IU/ml (NIL subtracted)	Suspected TB	132	2.525	5.5725	Single-Positive	1	1.14	0	-1.385	0.166667	1
TB1 IU/ml (NIL subtracted)	Suspected TB	132	2.525	5.5725	Stringent	13	2.02	3.9	-0.505	0.166667	1
TB1 IU/ml (NIL subtracted)	Single-Positive	1	1.14	0	Stringent	13	2.02	3.9	0.88	0.166667	1
TB2 IU/ml (NIL subtracted)	Control	1525	1.88	4.17	Suspected TB	132	2.755	5.4075	0.875	0.006092	0.036554
TB2 IU/ml (NIL subtracted)	Control	1525	1.88	4.17	Stringent	13	3.86	7.45	1.98	0.015801	0.094807
TB2 IU/ml (NIL subtracted)	Suspected TB	132	2.755	5.4075	Stringent	13	3.86	7.45	1.105	0.145259	0.871556
TB2 IU/ml (NIL subtracted)	Control	1525	1.88	4.17	Single-Positive	1	5.62	0	3.74	0.166667	1
TB2 IU/ml (NIL subtracted)	Suspected TB	132	2.755	5.4075	Single-Positive	1	5.62	0	2.865	0.166667	1
TB2 IU/ml (NIL subtracted)	Single-Positive	1	5.62	0	Stringent	13	3.86	7.45	-1.76	0.166667	1
TB2 minus TB1	Control	1525	0.03	0.56	Suspected TB	132	0	0.4125	-0.03	0.020679	0.124076
TB2 minus TB1	Suspected TB	132	0	0.4125	Single-Positive	1	4.48	0	4.48	0.064161	0.384964
TB2 minus TB1	Control	1525	0.03	0.56	Single-Positive	1	4.48	0	4.45	0.099436	0.596615
TB2 minus TB1	Single-Positive	1	4.48	0	Stringent	13	0.07	1.19	-4.41	0.152602	0.915612
TB2 minus TB1	Control	1525	0.03	0.56	Stringent	13	0.07	1.19	0.04	0.166667	1
TB2 minus TB1	Suspected TB	132	0	0.4125	Stringent	13	0.07	1.19	0.07	0.166667	1
